# Solution Structure of the Broad-Spectrum Bacteriocin Garvicin Q

**DOI:** 10.3390/ijms26167846

**Published:** 2025-08-14

**Authors:** Tyler Mallett, Tess Lamer, Tamara Aleksandrzak-Piekarczyk, Ryan T. McKay, Karizza Catenza, Clarissa Sit, Jan K. Rainey, Kaitlyn M. Towle-Straub, John C. Vederas, Marco J. van Belkum

**Affiliations:** 1 Department of Chemistry, University of Alberta, Edmonton, AB T6G 2G2, Canada; mallett1@ualberta.ca (T.M.); tlamer@ualberta.ca (T.L.); rmckay@ualberta.ca (R.T.M.); karizza@ualberta.ca (K.C.); john.vederas@ualberta.ca (J.C.V.); 2 Institute of Biochemistry and Biophysics, Polish Academy of Sciences, 02-106 Warsaw, Poland; tamara@ibb.waw.pl; 3 Department of Chemistry, Saint Mary’s University, Halifax, NS B3H 3C3, Canada; clarissa.sit@smu.ca; 4 Department of Biochemistry & Molecular Biology, Department of Chemistry, School of Biomedical Engineering, Dalhousie University, Halifax, NS B3H 4R2, Canada; jan.rainey@dal.ca; 5 Department of Physical Sciences, MacEwan University, Edmonton, AB T5J 2P2, Canada; towlek5@macewan.ca

**Keywords:** bacteriocin, NMR, antibiotic, garvicin Q, Man-PTS, SUMO, AlphaFold

## Abstract

Class IId bacteriocins are linear, unmodified antimicrobial peptides produced by Gram-positive bacteria, and often display potent, narrow-spectrum inhibition spectra. Garvicin Q (GarQ) is a class IId bacteriocin produced by the lactic acid bacterium *Lactococcus garvieae*. It stands out for its unusual broad-spectrum antimicrobial activity against various bacterial species, including *Listeria monocytogenes*, *Pediococcus pentosaceus*, *Carnobacterium maltaromaticum*, *Enterococcus faecalis*, and *Lactococcus* spp. Its protein target is the mannose phosphotransferase system (Man-PTS) of susceptible bacterial strains, though little is known about the precise molecular mechanism behind GarQ’s unusual broad spectrum of activity. In this work, ^13^C- and ^15^N-labelled GarQ was recombinantly produced using our previously described “sandwiched” protein expression system in *Escherichia coli*. We also developed a protocol to purify a uniformly labelled sample of the small ubiquitin-like modifier His_6_-SUMO, which is produced as a byproduct of the expression procedure. We demonstrated its use as a “free” protein standard for 3D NMR experiment calibrations. The GarQ solution structure was solved using triple-resonance nuclear magnetic resonance (NMR) spectroscopy and was compared with the structures of other Man-PTS-targeting bacteriocins. GarQ adopts a helix–hinge–helix fold, which is contrary to its structural predictions according to AlphaFold 3.

## 1. Introduction

There is a rapidly emerging need to develop novel antibiotics to slow the progression of antimicrobial resistance (AMR). Recent estimates suggest AMR-related infections contribute to 700,000 deaths annually, placing a significant burden on global healthcare systems [1]. Bacteriocins are ribosomally synthesized antibacterial peptides produced by bacteria and are a promising class of compounds to help address the challenges associated with AMR. Some bacteriocins, such as the lantibiotic nisin A, are widely used in food preservation, while others are being investigated as potential therapeutics due to their potent activity against bacterial pathogens [2]. Notably, bacteriocins derived from lactic acid bacteria (LAB) are also “Generally Recognized as Safe” (GRAS) and exhibit minimal cytotoxicity [3]. The majority of bacteriocins from LAB are divided into two different classes. Class I bacteriocins consists of post-translationally modified peptides, such as nisin, whereas class II bacteriocins include small post-translationally unmodified peptides [4]. Many class II bacteriocins from LAB are so-called class IIa bacteriocins, which are linear, single peptides that contain a YGNG motif at the N-terminus, as well as at least one disulfide bridge. Two-peptide bacteriocins and leaderless bacteriocins are often termed class IIb and class IIc bacteriocins, respectively. The 3D structures of a number of these class II bacteriocins have been elucidated using NMR spectroscopy [4]. Class IId bacteriocins (linear, single peptides without a YGNG motif) represent a functionally diverse but relatively understudied group of bacteriocins from LAB with significant therapeutic potential [4,5]. Among these, garvicin Q (GarQ), produced by *Lactococcus garvieae*, stands out for its broad-spectrum antimicrobial activity towards Gram-positive bacteria, including *Listeria monocytogenes*, *Pediococcus pentosaceus*, *Carnobacterium maltaromaticum*, *Enterococcus* spp., *Lactococcus* spp., and *Lactobacillus* spp. [6,7]. GarQ is synthesized as a 70-amino acid propeptide with a 20-residue N-terminal leader that is cleaved during maturation and export from the host organism, revealing a mature 50-residue bacteriocin [6]. GarQ remains stable over wide pH and temperature ranges while retaining sub-micromolar antimicrobial activity [6].

The receptor for GarQ on target cells is the mannose phosphotransferase system (Man-PTS) [7,8]. Man-PTS is a transmembrane protein complex used by some bacteria for the import and phosphorylation of monosaccharides, including mannose [9,10]. Man-PTS has been shown to serve as the receptor for class IIa bacteriocins, which are produced by LAB and contain a YGNG motif, as well as for dozens of unrelated bacteriocins with diverse structures and antimicrobial activities, making it one of the most widely recognized targets for bacteriocin activity [11]. Recent structural investigations using cryo-electron microscopy (cryo-EM) have demonstrated that Man-PTS-targeting bacteriocins, such as pediocin PA-1 (PedA—class IIa) and lactococcin A (LcnA—class IId), bind the Man-PTS complex between the Core and Vmotif transmembrane domains [12,13]. These interactions are thought to facilitate membrane pore formation, thereby dissipating the proton motive force and leading to cell death. Although PedA and LcnA both bind the Man-PTS receptor, they share minimal sequence similarity and interact with overlapping but non-identical regions of the IIC domains. Structural studies have shown that both peptides form membrane pores through the insertion of an amphiphilic C-terminal α-helix—a domain that is thought to play a role in target cell specificity. However, their distinct N-terminal β-sheet domains contribute to differences in receptor recognition and binding specificity [12,13,14]. Notably, the β-sheet region of LcnA is longer than that of PedA and is involved in IIC domain binding at the region-α subdomain in *L. lactis*. [12,13,15]. Differences in bacterial susceptibility to individual bacteriocins may arise from subtle variations in the Core domains of sensitive species rather than distinct mechanisms of pore formation [11,16].

In this study, we determined the NMR solution structure of GarQ to further understand the structural differences between bacteriocin peptides that exert their antimicrobial effects by binding to Man-PTS. Isotopically labelled (^13^C and ^15^N) GarQ was isolated using our previously reported “sandwiched” fusion protein system [17,18]. We also developed a protocol to easily isolate and purify the isotopically labelled byproduct His_6_-SUMO in order to provide a cost-effective reference standard for future 3D NMR applications [17,18,19]. The NMR-derived structure of GarQ was compared to the Man-PTS-bound structures of PedA and LcnA, as well as to other structures of Man-PTS-targeting bacteriocins, including microcin E492 (MccE492—a bacteriocin from Gram-negative bacteria) [20], leucocin A (LeuA—class IIa) [21,22], carnobacteriocin B2 (CbnB2—class IIa) [22], sakacin P (SakP—class IIa) [23], curvacin A (CurA—class IIa) [24], and to the AlphaFold 3 (AF3) GarQ structure [25]. The NMR-derived GarQ solution structure was found to adopt a helix–hinge–helix fold, similar to that of curvacin A [24]. GarQ differs significantly from the published structures of LcnA, PedA, LeuA, and SakP, as well as the predicted AF3 GarQ structure, which all contain N-terminal β-sheets and C-terminal α-helices [12,13,21,22,23,25]. The α-helical content between GarQ and LcnA was examined in trifluoroethanol (TFE) using circular dichroism (CD) experiments to validate the increased α-helicity observed in the GarQ NMR solution structure relative to the LcnA cryo-EM receptor-bound structure [13]. We found that GarQ has an increased α-helical content in TFE relative to LcnA.

## 2. Results

### 2.1. Isolation of [U-^13^C/^15^N]-Labelled GarQ and His_6_-SUMO

*E. coli* cells containing the pSPIH6 plasmid were grown in uniformly ^13^C- and ^15^N-labelled Spectra 9 media, and the GarQ protein was overexpressed as described previously [16,18]. A total of 4 mgs of GarQ was used to prepare a 1.0 mM solution in 1:1 H_2_O:2,2,2-trifluoroethanol-d_2_ (d_2_-TFE) at pH 7 for NMR experimentation, according to previously reported circular dichroism (CD) experiments that demonstrate a shift from disorder to structure at this concentration of d_2_-TFE [11]. Labelled His_6_-SUMO and Intein-CBD-His_6_ are byproducts of using the SPI expression protocol in isotopically labelled media, and His_6_-SUMO was isolated from Intein-CBD-His_6_ using a chitin affinity column (Supplementary Figure S1).

### 2.2. SUMO NMR Structural Elucidation

His_6_-SUMO was concentrated and exchanged to a 90:10 H_2_O/D_2_O phosphate-buffered solution at pH 6.5 for NMR experiments. His_6_-SUMO was found to be well-structured, as evidenced by amide chemical shift dispersion in the ^1^H-^15^N-HSQC spectra (Supplementary Figure S2). A standard suite of biological NMR experiments was conducted, and the chemical shift and NOE data were assigned. The His_6_-SUMO ensemble coordinates were deposited in the Protein Data Bank (PDB) (accession 9OIU), and all chemical shift lists were deposited in the Biological Magnetic Resonance Data Bank (BMRB) (accession 53021).

### 2.3. GarQ and LcnA Circular Dichroism

GarQ CD spectra in differing concentrations of H_2_O:TFE have been published previously and demonstrate that GarQ is structured in 50% TFE [11]. We expressed and purified LcnA, which is another type IId bacteriocin, using the method described by van Belkum et al. [16]; we examined its structure in a variety of H_2_O:TFE solvent compositions for comparison with GarQ. We measured the molar ellipticity between wavelengths of 250 and 185 nm, and the data were plotted. The α-helical content was determined using Equation (1) [11,26]. We compared the LcnA CD data to the previously published GarQ CD data and found that the approximate α-helicity of GarQ and LcnA in 50% TFE was ~36% and ~21%, respectively (Supplementary Figure S3).

(1) $$\%\;\alpha -helix=\left(\frac{-\left[\theta_{222}\right]+3000}{39000}\right)\times 100\%$$

### 2.4. GarQ NMR Structural Elucidation

**Figure 1:**
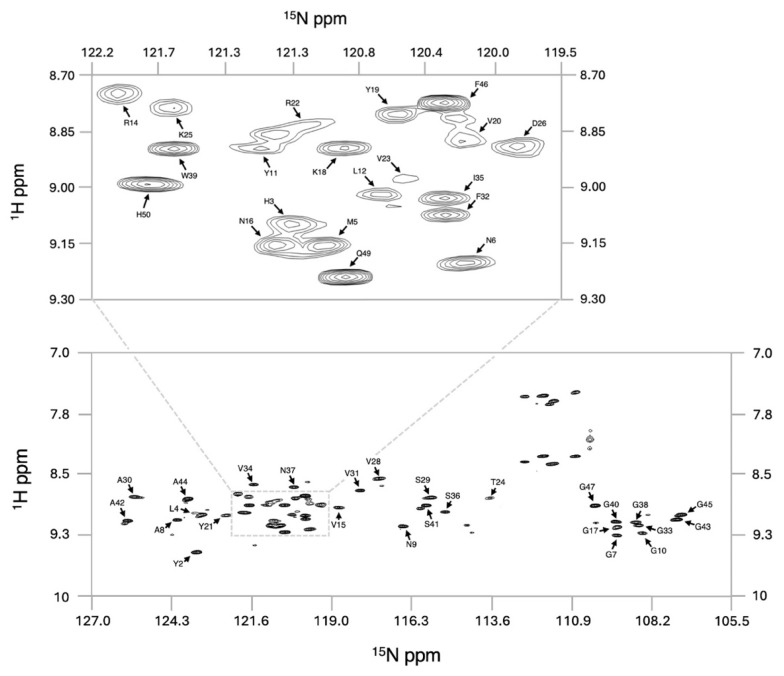
^1^H-^15^N-HSQC spectrum of GarQ. Assigned full spectrum (**bottom**) and expansion of a central region (**top**). Assignment labels highlight the amide NH chemical shift of each amino acid.

To investigate the solution structure of GarQ, we prepared a uniformly ^13^C/^15^N isotopically labelled GarQ sample, which was found to adopt a defined structure in 50% d_2_-TFE in water at pH 7, as evidenced by significant amide chemical shift dispersion in the GarQ ^1^H-^15^N-HSQC spectrum (Figure 1).

**Figure 2:**
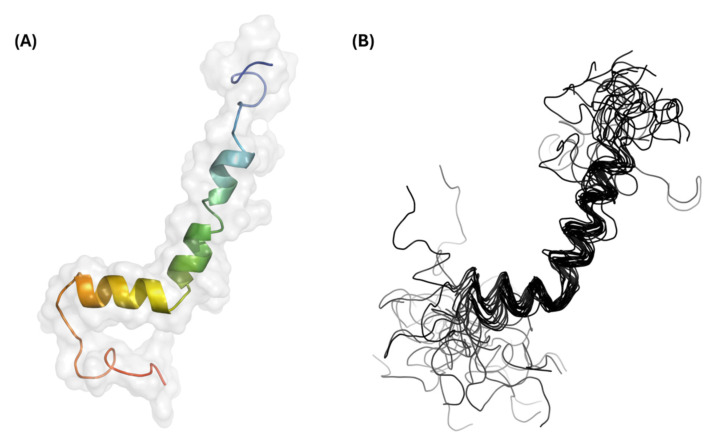
Three-dimensional structural results for GarQ. (**A**) Structural model and surface representation of the lowest-energy GarQ structure. The coloring is depicted as a rainbow gradient whereby the N-terminus is colored blue and the C-terminus is colored red. (**B**) Structural ensemble containing the 20 lowest-energy GarQ structures.

Additional NMR experiments were completed to determine the backbone and sidechain chemical shifts, achieving an overall assignment completeness of 71%. In total, 1000 structures were generated during each round of structure calculations, and the 20 lowest-energy models were combined to produce the GarQ protein ensemble (Figure 2). The solution structure of GarQ was obtained after 34 rounds of refinements and structure calculations. Our structure has no geometric outliers and no Ramachandran outliers in the structured-core region comprising of residues 18–37 (Supplementary Figure S4). The GarQ solution structure resides in the 78th and 97th percentiles for backbone and sidechain torsion angles, respectively, when compared to all currently published structures in the PDB. The overall structure root mean squared deviation (RMSD) value for the deposited protein ensemble was 0.18 ± 0.03 Å. Summarized NMR structural statistics are included in Table 1. The protein ensemble coordinates have been deposited in the PDB (accession 9OIL) and the chemical shift lists have been deposited in the BMRB (accession 53020).

### 2.5. GarQ Structure Comparisons to Cryo-EM Structures of Man-PTS-Bound Bacteriocins

**Figure 3:**
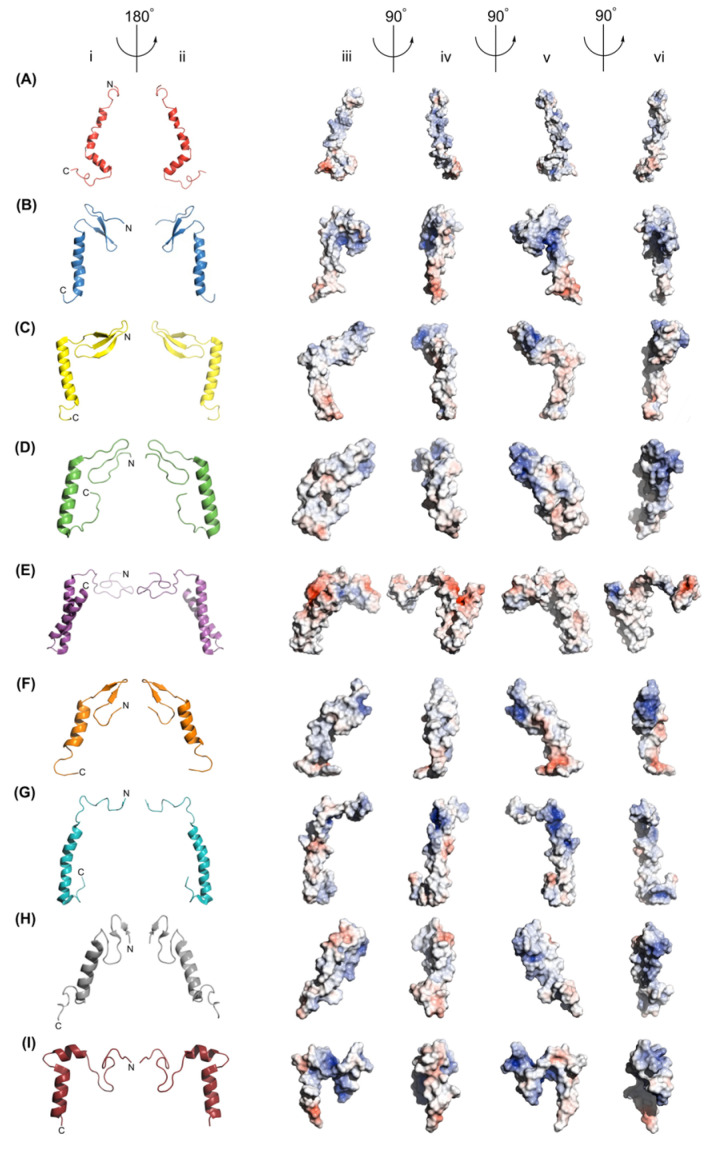
Structural model representations of (**A**) the GarQ NMR structure, (**B**) the GarQ AlphaFold 3 structure, (**C**) the LcnA cryo-EM structure, (**D**) the PedA cryo-EM structure, (**E**) the MccE492 cryo-EM structure, (**F**) the LeuA NMR structure, (**G**) the CbnB2 NMR structure, (**H**) the SakP NMR structure, and (**I**) the CurA NMR structure, as shown in columns (i) and (ii). In all cartoon images, the N-terminus is positioned at the top and to the right of the centre. Electrostatic surface representations of all bacteriocins are shown in columns (iii), (iv), (v), and (vi). Structures in columns (i) and (iii) share the same orientation, as do those in columns (ii) and (v). Positively charged surface areas are shown in blue, negatively charged areas are shown in red, and neutral regions are shown in grey. Arrows and angle indicators show the axes and directions of rotation between views.

The GarQ solution structure is depicted in Figure 3, which also includes the structures of several other Man-PTS-targeting bacteriocins. We found that GarQ has well-resolved N- and C-terminal α-helices with RMSD values of 0.25 Å and 0.09 Å, respectively. An unstructured hinge region separates the two α-helices. The GarQ helix–hinge–helix fold is not observed in the class IId bacteriocin LcnA [13], the class IIa bacteriocin PedA [12], or the class IIa bacteriocin MccE492 [20], all of which were resolved using cryo-EM while bound to the Man-PTS (Figure 3). LcnA and PedA both feature N-terminal β-sheets and a C-terminal α-helix separated by an unstructured hinge region [12,13]. It should be noted that class IIa bacteriocins, such as PedA, are characterized by a disulfide bridge in the N-terminal domain following the YGNG motif, which may confer structural stability of class IIa bacteriocins [4]. In contrast, MccE492 has a random coiled N-terminus and two membrane-spanning α-helices [20]. We compared the structural overlay RMSD between portions of the GarQ solution structure to the respective regions of the LcnA, PedA, and MccE492 cryo-EM structures; the results are included in Table 2.

**Figure 4:**
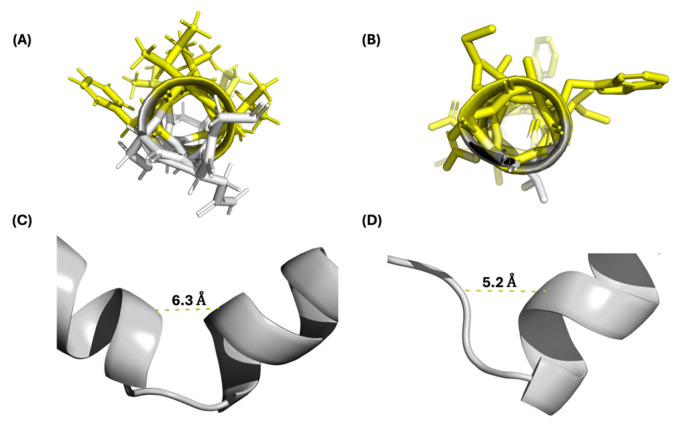
(**A**) GarQ C-terminal α-helix; hydrophobic amino acids highlighted in yellow. (**B**) LcnA C-terminal α-helix; hydrophobic amino acids highlighted in yellow. (**C**) GarQ hinge region. (**D**) LcnA hinge region. The distances shown were measured from the α-carbons of GarQ residues 23 to 29 and LcnA residues 28 to 33.

The experimentally derived structures of the class IId bacteriocins GarQ and LcnA were found to have an overlapping structural RMSD of 3.09 Å and a sequence similarity of 44.4%. GarQ and LcnA share an anionic and amphiphilic C-terminal α-helix, which has a structural alignment RMSD of 0.26 Å (Figure 4A,B and Figure S5). The N-terminal α-helix of GarQ and the β-sheet of LcnA were found to have a structural alignment RMSD of 1.13 Å. GarQ and LcnA both feature an unstructured hinged region that separate the N- and C-terminal structured regions. The distance of the hinged regions was measured between the α-carbons of GarQ residues 23 to 29 and LcnA residues 28 to 33, which were found to be 6.3 Å and 5.2 Å, respectively (Figure 4C,D).

PedA and MccE492 were found to have an overall structural overlay RMSD with GarQ of 2.91 Å and 2.44 Å, respectively. The C-terminal α-helices of GarQ and PedA were in best agreement when compared to all other bacteriocin structures in this study, with an RMSD of 0.25 Å, despite a poor sequence similarity of 32.2%. The GarQ C-terminal α-helix had a comparable structure RMSD of 0.3 with residues G28-G52 of MccE492, which comprises its C-terminal α-helix. The GarQ N-terminus shared a structural alignment RMSD with MccE492 residues 5–28 and PedA residues 2–19 of 1.19 and 3.17, respectively.

### 2.6. GarQ Structure Comparisons to NMR Solution Structures of Man-PTS-Targeting Bacteriocins

We compared the GarQ NMR structure with those of other Man-PTS-targeting bacteriocins that were derived from NMR experiments, including LeuA [21,22], CbnB2 [22], SakP [23], and CurA [24], all of which are class IIa bacteriocins. Of these, LeuA, CbnB2, and SakP were resolved in a 9:1 TFE:H_2_O environment in acidic conditions (pH 2.8). CurA was resolved in a 9:1 H_2_O:D_2_O environment containing 350 mM dodecyl phosphocholine micelles under acidic conditions (pH 2.8). LeuA and SakP adopt N-terminal β-sheets and C-terminal α-helices, which exhibit an overall structure alignment RMSD with GarQ of 2.51 Å and 2.86 Å, respectively. GarQ was found to have a structurally conserved C-terminal structure with LeuA and SakP, with RMSD values of 0.57 Å and 0.49 Å, respectively.

CbnB2 and CurA, unlike LeuA and SakP, both adopt strictly α-helical secondary structures. CbnB2 contains a single α-helix, comprising residues 17–42 that was found to have an overall structure alignment RMSD with GarQ of 3.28 Å. We overlaid the N- and C-terminal α-helices of GarQ with residues 17–42 of CbnB2 and found structural overlay RMSDs of 0.93 Å and 0.44 Å, respectively. CurA, much like GarQ, adopts a helix–hinge–helix fold [24]. CurA and GarQ share N- and C-terminal α-helix structural overlay RMSDs of 0.41 Å and 0.58 Å, respectively, with an overall structural RMSD of 2.21 Å. Of all the bacteriocin structures included in this study, CurA was found to have the best N-terminal secondary structure overlay with GarQ.

### 2.7. Comparison to the GarQ AlphaFold 3 Model

We also used AlphaFold 3 to generate a predicted structure for GarQ based on the amino acid sequence [25]. The AF3-predicted GarQ structure contains an N-terminal β-sheet and C-terminal α-helix from amino acid residues 2–27 and 29–48, respectively. We found the experimentally derived and predicted GarQ structures to have an overall structure alignment RMSD of 2.13 Å, which is the best result of all the bacteriocin structures used in this study. The N- and C-terminal secondary structure portions between the experimental and theoretical GarQ structures were found to have a structural overlay RMSD of 2.7 Å and 0.59 Å, respectively.

## 3. Discussion

Class IId bacteriocins produced by LAB are of significant interest for their potent antimicrobial characteristics. However, many are limited by a narrow activity spectrum [4]. We report the solution structure of garvicin Q, which represents one of the few cases of a class IId bacteriocin with potent activity against a broad spectrum of bacterial strains [6,7]. GarQ was found to have an N-terminal α-helix from residues 18–26 and a C-terminal α-helix from residues 29–37, with an overall structure RMSD of 0.18 ± 0.03 Å. GarQ is unusual in that it appears to adopt an exclusively α-helical fold featuring a helix–hinge–helix structure, similar to what is observed for curvacin A [24]. Like many Man-PTS-targeting bacteriocins, GarQ was predicted to contain an N-terminal anti-parallel β-sheet and a C-terminal α-helix [11]. This type of bacteriocin fold is observed in the published structures of LcnA [13], PedA [12], LeuA [21,22], and SakP [23]. The structures of LcnA and PedA are a product of cyro-EM investigations of the bacteriocins bound to Man-PTS, where it was found that the C-terminal α-helix inserts between the Man-PTS Core and Vmotif domains [12,13]. The N-terminal β-sheet was shown to be involved in receptor docking by interacting with the Man-PTS region-α [12,13]. LeuA and SakP were elucidated in solution using NMR spectroscopic techniques in both TFE and dodecyl phosphocholine micelles; they are proposed to elicit bactericidal activity through a similar mechanism [21,22,23].

**Figure 5:**
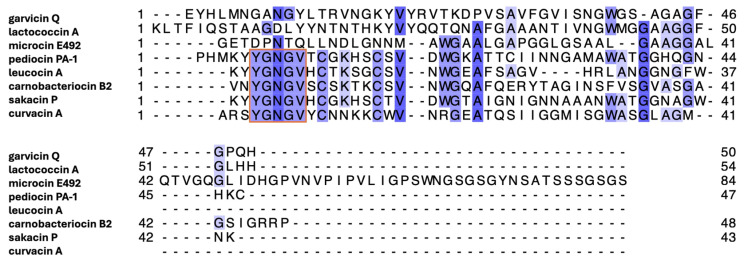
Multiple sequence alignment of bacteriocins described in this study. Orange box indicates the conserved YGNGV motif of class IIa bacteriocins. The most conserved residues are colored dark blue, whereas the least conserved residues are colored light blue. The multiple sequence alignment was generated using the UniProt Align online tool.

There are also structures of Man-PTS-targeting bacteriocins that adopt exclusively α-helical secondary structures, including MccE492 [20], CbnB2 [22], and CurA [24]. The structures of these bacteriocins were elucidated using different techniques, with MccE492 being bound to Man-PTS as a glutathione S-transferase fusion protein and resolved using cryo-EM [20]. Structures for CbnB2 and CurA were resolved using solution NMR experiments in d_2_-TFE and dodecyl phosphocholine micelles, respectively [22,24]. GarQ appears to reside within the α-helical-type bacteriocins, with excellent structural overlap (RMSD < 1 Å) of both its N- and C-terminal α-helices with the respective regions of CbnB2 and CurA, despite little to no sequence identity (Table 2, Figure 5).

GarQ had the highest degree of sequence identity and similarity with the class IId bacteriocin LcnA (Figure 5). The GarQ and LcnA C-terminal α-helices overlapped well, with a backbone RMSD of 0.26 Å. However, GarQ and LcnA differ significantly in N-terminal secondary structure, as evidenced by the poor N-terminal structural overlay RMSD of 1.13 Å. The GarQ C-terminal α-helix was found to be in good agreement with all Man-PTS-targeting bacteriocins used in this study. The best C-terminal overlay was with PedA (0.25 Å) and the worst was with CurA (0.58 Å), despite the low sequence identities [12,24]. When the GarQ N-terminal α-helix was overlaid with the respective N-terminal structured cores of the previously elucidated bacteriocins, it was CurA that was in best agreement. with an overlaid backbone RMSD of 0.41 Å.

A recent report has highlighted potential significant differences between the AlphaFold 3-predicted and experimentally derived structures of biological molecules [27]. For GarQ, the experimentally derived structure deviates significantly from the AlphaFold 3-generated GarQ structure. GarQ is predicted by AlphaFold 3 to have an N-terminal β-sheet and C-terminal α-helix, similar to what is observed for LcnA [13]. When compared, the experimental and predicted GarQ structures have an overlay RMSD of 2.13 Å and feature a moderately conserved C-terminal α-helix with a structure overlay RMSD of 0.59 Å. Notably, the AlphaFold 3-predicted structure of GarQ showed the poorest alignment of the C-terminal secondary structure compared to all Man-PTS-targeting bacteriocins examined in this study (Table 2). However, peptide structures are dependent on solvent environments. Linear, unmodified peptides are generally accepted to be randomly coiled in solution; this is clearly observed experimentally using circular dichroism with both GarQ [11] and LcnA (Supplementary Figure S3), where a transition from random coil to α-helical occurs as the solvent transitions from pure water to a mixture of water with structure-inducing TFE. It is therefore unclear how the solvent plays a role in AlphaFold 3 structural predictions of peptides, as most NMR solution structures of linear peptides deposited in the PDB were conducted in a non-water solvent system. Since it is known that fluorinated alcohol/H_2_O co-solvent compositions for NMR experimentation tend to favour the formation of α-helices [28], we were interested in examining the CD spectra of LcnA in solution and comparing these results to the published CD spectra of GarQ to help validate our findings [11]. In 50% TFE/H_2_O, we found the approximate α-helicity of GarQ and LcnA to be ~36% and ~21%, respectively. The observed difference in CD-derived α-helical structure between the two class IId bacteriocins may be a result of the differing N-terminal secondary structures observed between the cryo-EM LcnA model and our solution GarQ model.

The experimental GarQ and LcnA structures share common features, including an anionic C-terminus, an amphiphilic C-terminal α-helix, and unstructured kinks between their N- and C-terminal structural regions. The structural similarity observed in the C-terminal α-helices suggests that GarQ also interacts with the Man-PTS Core and V-motif domains, similar to what has been previously demonstrated for LcnA [13]. It remains unclear whether the GarQ N-terminus, with an α-helical rather than β-sheet structure, interacts with LcnA’s reported binding site of the Man-PTS region-α, particularly since Man-PTS mutations that are not in the vicinity of the LcnA binding site result in resistance to both LcnA and GarQ [16]. However, it cannot be ruled out that the binding of GarQ to Man-PTS induces an N-terminal β-sheet structure in GarQ. It should be stressed that direct comparisons of GarQ to previously reported structures are limited by differing experimental conditions and may reflect different structural states (e.g., free vs. receptor-bound), as has been explained above. The difference in N-terminal secondary structure may also contribute to the broad activity spectrum observed for GarQ. It has been shown previously that single amino acid substitutions in the N-terminal part of GarQ reduced the antimicrobial spectrum of GarQ, resulting in a narrow-spectrum variant targeting only a subset of strains [11]. In contrast, mutations in the central or C-terminal region had no such effect. Further studies into GarQ-Man-PTS receptor docking and bactericidal mechanisms are required to validate this hypothesis.

The low sequence identity and high structural conservation observed in the C-terminal α-helical domain of GarQ and the other class IId bacteriocins discussed here further underscores the idea that antimicrobial activity in these class IId bacteriocins is driven more by structural topology than by primary sequence, as reported in previous structural studies [12,13,14,15]. This suggests that future peptide engineering efforts should focus on preserving the amphipathic and anionic properties of the C-terminal helix. Furthermore, the observed variability in the secondary structures of the N-terminal domain, along with the previous single amino acid substitution studies for GarQ, indicates that this region may be a key determinant of the spectrum of activity for these bacteriocins, making it an attractive site for engineering variants with broadened or narrowed antimicrobial activity. Future studies investigating the structural basis of N-terminal interactions with the Man-PTS receptor could inform the design of synthetic analogues with enhanced stability, optimized binding, and tunable activity profiles.

While configuring the required 3D-NMR experiments necessary to elucidate the GarQ solution structure, it became apparent that a labelled 3D-NMR protein standard would be beneficial to expedite instrument optimization and to ensure spectrometer consistency. Additionally, a reference data set may help validate the hardware and software configurations required to calculate NMR solution structures [29]. Current prices of commercially available 3D-NMR protein standards are a significant expenditure for many biological NMR spectroscopists.

Small Ubiquitin-like Modifier (SUMO), which is a ubiquitin homologue, shares many of the same advantages as ubiquitin. SUMO is a defined globular protein that remains intact over large environmental and temperature ranges, which is helpful for fusion-protein expression, as well as being an applicable complementary 3D-NMR protein standard produced as a byproduct of the “sandwiched” SUMO-peptide-Intein expression procedure (Supplementary Figures S6 and S7) [17,18,30,31]. A 1L fermentation of *E. coli* with Spectra 9 ^13^C, ^15^N-media has been previously demonstrated to afford 2 mg of a desired ~5000 Da peptide [18]. For each expression using the “sandwiched” SPI technology, approximately 6.6 mg of the labelled His_6_-SUMO protein will be produced as a byproduct of the SPI purification procedure [18]. When compared with the commercial market prices of ^13^C/^15^N-Ubiquitin, the production and isolation of ^13^C/^15^N-His_6_-SUMO has a significant financial benefit.

## 4. Materials and Methods

### 4.1. General Method Information

Commercially available reagents were purchased from Bio-Rad Laboratories (Mississauga, ON, Canada), Cambridge Isotope Laboratories (Tweksbury, MA, USA), Chem-Impex International Inc. (Wood Dale, IL, USA), Sigma-Aldrich Canada (Oakville, ON, Canada), or Thermo Fisher Scientific (Ottawa, ON, Canada). All commercially purchased reagents were used without further purification. Deionized water was produced and collected from a Milli-Q reagent water filtration system (Millipore Co., Milford, MA, USA) and was used without further purification. Mass spectra were obtained using the Perspective Biosystems Voyager™ Elite matrix-assisted laser desorption ionization time of flight mass spectrometer (MALDI-TOF MS) (Thermo Fisher Scientific) Sinapinic acid was used as the matrix for all MS samples. MS spectra were analyzed using the Agilent Mass Hunter Qualitative Analysis Software Package (version B.03.01 SP3, Agilent Technologies, Santa Clara, CA, USA). Semi-preparative HPLC was carried out using an Agilent chromatograph (Agilent Technologies) with a 1260 Infinity II quaternary pump, diode array detector, fraction collector, and manual injector fitted with a 500 μL sample loop. A Vydac 219TP Diphenyl, 5 μ, 10 × 250 mm column was used (Canadian Life Science, Peterborough, ON, Canada). All HPLC solvents were purchased as HPLC grade and were filtered through a Millipore filtration system under vacuum before use.

### 4.2. Expression, Purification, and NMR Sample Preparation of GarQ

The expression and purification of GarQ using the pSPIH6 expression system in *E. coli* BL21 (DE3) have been described previously [16,18]. In this study, [U-^13^C/^15^N]-GarQ was produced by culturing *E. coli* containing the pSPIH6-GarQ plasmid in isotopically labelled medium (Spectra 9 ^13^C,^15^N liquid media) (Cambridge Isotope Laboratories) supplemented with ampicillin (100 µg/mL) in order to facilitate multidimensional NMR experiments. Following expression and cell lysis, the fusion protein was purified using Ni-NTA affinity chromatography and was cleaved with SUMO protease; then, the cleaved GarQ was isolated as described in the original protocol. GarQ was lyophilized and redissolved in an acetonitrile/water solution with 0.01% trifluoroacetic acid. The GarQ peptide was subsequently purified using a semi-preparative HPLC with 400 μL injection volumes, as previously described [16]. MALDI-TOF MS was used to confirm the identity of the purified GarQ sample. The purified sample was again lyophilized to obtain approximately 4 mg of pure peptide. A 1.0 mM GarQ solution was prepared in 300 μL of 1:1 H_2_O:2,2,2-trifluoroethanol-d_2_ (d_2_-TFE) at pH 7, which was transferred to a 5 mm i.d. D_2_O-matched Shigemi NMR tube (BMS-005 Varian type, Sigma Aldrich). A slight precipitate was observed immediately after peptide suspension; however, it settled to the bottom of the tube and did not interfere with NMR data acquisition. The sample remained stable for several months at 4 °C.

### 4.3. His_6_-SUMO Purification and NMR Sample Preparation

His_6_-SUMO was obtained in the second Ni-NTA elution fraction according to the “sandwiched” SPI purification procedure [18] and was separated from intein-CBD-His_6_ using affinity chromatography. Chitin beads (5 mL) (New England Biolabs, Ipswich, MA, USA) were added to the elution solution, which was gently shaken at 4 °C for one hour. After this time, a third fritted column was used, and His_6_-SUMO was collected in the column flow-through fraction. His_6_-SUMO was dialyzed to lower the imidazole concentration and to exchange the buffer (10 mM NaH_2_PO_4_, 50 mM imidazole, pH 7). His_6_-SUMO was then concentrated using an Amicon^®^ Ultra Centrifugal Filter with a 3 kDa molecular weight cut-off (Sigma-Aldrich Canada). The buffer was exchanged to 90:10 H_2_O/D_2_O, 10 mM NaH_2_PO_4_, 0.02% NaN_3_, pH 6.5, which is suitable for NMR experiments. The sample was concentrated to 650 μL to obtain a 1.0 mM solution, before being transferred to a 5mm i.d. NMR tube and stored at 4 °C between NMR experiments.

### 4.4. NMR Experimental Parameters

Biological NMR experiments were set up and conducted using a Varian INOVA VNMRS four-channel 600 MHz spectrometer or a Varian VNMRS four-channel, dual-receiver 700 MHz spectrometer (Palo Alto, CA, USA). Additional NMR experiments were performed using a Bruker AVIIID 800 MHz spectrometer (Billerica, MA, USA). Multi-dimensional NMR experiments used to elucidate the structure of GarQ include ^1^H-^15^N-HSQC [32], HNCACB [33], ^15^N-TOCSY-HSQC, ^15^N-HSQC-NOESY [34] (VNMRS 700, MHz spectrometer, Varian), HCCH-TOCSY [35], and ^13^C-HSQC-NOESY [36] (AVIIID 800 MHz spectrometer, Bruker).

The suite of collected NMR experiments used to elucidate the structure for His_6_-SUMO includes ^1^H-^15^N-HSQC [32], ^1^H-^13^C-HSQC [36], HNCO [37], HN(CA)CO [38], HNCACB [33], CBCA(CO)NNH [39,40], HNCA [41,42], HN(CO)CA [43], ^15^N-TOCSY-HSQC, ^15^N-HSQC-NOESY [34] (INOVA 600 MHz spectrometer, Varian), HCCH-TOCSY [35], and ^13^C-HSQC-NOESY [36] (AVIIID 800 MHz spectrometer, Bruker). All experiments were referenced using a water proton chemical shift of ~4.8 ppm (27 °C calibrated). Chemical shifts for most atoms in His_6_-SUMO were assigned, excluding sidechain heteroatoms and aromatic systems. The calculated His_6_-SUMO structures were compared to previous reports of SUMO solution NMR structures [44]. NMR experiments were not conducted to elucidate the chemical shifts in sidechain aromatic atoms. Detailed experimental parameters, including the number of points collected, acquisition times, sweep widths, experimental temperatures, and chemical shift lists, are provided in the Supplementary Information.

### 4.5. NMR Data Assignment and Structure Calculations

All protein NMR data sets were analyzed and assigned using CcpNMR (version 3.2.12, Leicester, UK) [45]. Structure calculations were completed using Xplor-NIH (version 3.9.8, Bethesda, MD, USA) [46,47]. NOE distance restraints, torsion angle restraints, and dihedral restraints were compiled from TALOS-N (version 4.1, Bethesda, MD, USA) [48]. A total of 1000 structures were calculated during each round of calculation, of which the 20 lowest-energy structures were compiled into the final protein ensemble. In total, 34 rounds of calculations were completed to obtain the final structure for GarQ. Distance restraint and dihedral-angle violations were analyzed using TCL/TK scripts and validation reports generated by the wwPDB NMR Structure Validation Report Service [49,50]. Molecular structures were visualized using PyMol, and the electrostatic surface depictions were produced using the APBS Electrostatics Plugin for Pymol [51]. Hydropathic character maps were generated in Pymol using the Kyte–Doolittle hydrophobicity scale [52]. Ramachandran maps and statistics were created using the RamPlot online service and the wwPDB Validation Report Service [53]. All overlayed structure statistics were generated with the online RCSB Pairwise Structure Alignment Tool [54], and the bacteriocin amino acid sequences were compared using the EMBL-EMBOSS server [55]. A multiple sequence alignment of all the bacteriocins discussed in this study was generated using the UniProt Align online tool [56]. The structure coordinates were deposited as PDB entries 9OIL (GarQ) and 9OIU (His_6_-SUMO). The compiled structural restraint data, chemical shift assignments, and peak lists were submitted and deposited as BMRB entries 53020 (GarQ) and 53021 (His_6_-SUMO).

## 5. Conclusions

Here, we report the solution structure of GarQ, which is a highly potent broad-spectrum type IId bacteriocin, which contains two α-helices separated by an unstructured hinge. The GarQ solution structure was compared with the reported structures of other Man-PTS-targeting bacteriocins and the AlphaFold 3 GarQ structure. The GarQ solution NMR structure lacks the N-terminal β-sheet found in many currently known Man-PTS-targeting bacteriocins, as well as that is predicted using AlphaFold 3. Instead, GarQ appears to adopt a helix–hinge–helix structure, similar to that of curvacin A. The presence of an N-terminal α-helix, rather than a β-sheet, may contribute to GarQ’s broad spectrum of activity. However, further studies are required to fully elucidate GarQ’s binding mechanism with the Man-PTS system.

We also report an expansion of the methodology for the previously described “sandwiched” SUMO–peptide–intein expression system. Here, we readily isolated fully labelled ^13^C,^15^N-His_6_-SUMO (His_6_-SUMO), which is a byproduct of ^13^C,^15^N-GarQ expression. We show that His_6_-SUMO can be utilized as a complementary protein standard for 3D-NMR experiments, which can be accessed at a fraction of the cost of current commercially available 3D-NMR standards. All experimental spectra, conditions, and methodologies are freely available and can be found in the Supplementary Information.

## Figures and Tables

**Table 1:** Structural statistics for GarQ. Data with an * were gathered using Xplor-NIH calculation statistics. Unmarked data were obtained from the wwPDB Structure Validation Service.

No. of Constraints	
Total No. of NOE constraints	225
Short-range	101
Medium-range ($1<\left|i-j\right|<5$)	63
Long-range ($\left|i-j\right|>5$)	10
Total No. of dihedral angle constraints	58
*φ*	25
*ψ*	25
*χ*	8
Constraint Violations	
Distance constraint violations	8
Dihedral-angle constraints	0
RMSD of the calculated protein structure	
* Whole structure (Å)	0.18 ± 0.03
A:18-A:26 (9) (Å)	0.25
A:29-A:37 (9) (Å)	0.09
Ramachandran statistics (%)	
Favoured regions	45
Allowed regions	2
Disallowed regions	1
Assignment statistics	
No. of assigned peaks	444
No. of unassigned peaks	197
Assignment completeness	71%

**Table 2:** Man-PTS-targeting bacteriocin structure and sequence comparisons relative to the GarQ solution structure. Structure overlay statistics and sequence comparisons were generated using the online RCSB Pairwise Structure Alignment Tool.

Bacteriocin	Whole Structure Overlay (Å)	Overlaid N- and C-Terminal Residues	N-Terminal Overlay (Å)	C-Terminal Overlay (Å)	Sequence Identity	Sequence Similarity	Method
garvicin Q (PDB: 9OIL)	-	G10-K25, P27-G38	-	-	-	-	NMR (d_2_-TFE/H_2_O)
AlphaFold 3 GarQ	2.13	G10-V23, P27-G45	2.7	0.59	100%	100%	Computational
lactococcin A (PDB: 8HFS)	3.09	L2-T27, N29-G48	1.13	0.26	35.2%	44.4	Cyro-EM
pediocin PA-1 (PDB: 7VLY)	2.91	H2-V19, D20-G39	3.17	0.25	23.7%	32.2%	Cryo-EM
microcin E492 (PDB: 7DYR)	2.44	P5-G28, G28-G52	1.19	0.3	13.8%	23%	Cyro-EM
leucocin A (PDB: 1CW6)	2.51	G6-N17, W18-A30	3.01	0.57	6.7%	9.3%	NMR (d_3_-TFE/H_2_O)
carnobacteriocin B2 (PDB: 1CW5)	3.28	N17-G42	0.93	0.44	26.3%	31.6%	NMR (d_3_-TFE/H_2_O)
sakacin P (PDB: 1OG7)	2.86	Y2-V16, W18-W33	1.02	0.49	16.1%	27.4%	NMR (d_3_-TFE/H_2_O)
curvacin A (PDB: 2A2B)	2.21	N15-S25, G28-G40	0.41	0.58	19.3%	28.1%	NMR (DPC/H_2_O)

## Data Availability

The GarQ chemical shift list and structure have been submitted to the BMRB under accession code 53020; the NMR-STAR file can be viewed at https://bmrb.io/author_view/53020_hy_xiajtwvr.str (accessed on 6 May 2025). NMR data for GarQ have been deposited in the PDB under accession number 9OIL and can be viewed at the following PDB DOI: https://doi.org/10.2210/pdb9OIL/pdb (accessed on 10 May 2025). The His_6_-SUMO chemical shift list and structure have been submitted to the BMRB under accession code 53021; the NMR-STAR file can be viewed at https://bmrb.io/author_view/53021_hy_jptamjeh.str (accessed on 18 April 2025). NMR data for His_6_-SUMO have been deposited in the PDB under accession number 9OIU and can be viewed at the following PDB DOI: https://doi.org/10.2210/pdb9OIU/pdb (accessed on 6 May 2025).

## Supplementary Materials

The following supporting information can be downloaded at https://www.mdpi.com/article/10.3390/ijms26167846/s1.

## Abbreviations

The following abbreviations are used in this manuscript:

AF3	AlphaFold 3
AMR	Antimicrobial Resistance
CBD	Chitin-Binding Domain
CbnB2	Carnobacteriocin B2
CD	Circular Dichroism
CurA	Curvacin A
GarQ	Garvicin Q
GRAS	Generally Recognized as Safe
His_6_-SUMO	Hexahistidine-tagged Small Ubiquitin-like Modifier
HPLC	High-Performance Liquid Chromatography
IPTG	Isopropyl-β-D-1-thiogalactopyranoside
LAB	Lactic Acid Bacteria
LcnA	Lactococcin A
LeuA	Leucocin A
Man-PTS	Mannose phosphotransferase system
MccE492	Microcin E492
MS	Mass Spectrometry
Ni-NTA	Nickel-Nitrilotriacetic acid resin
NMR	Nuclear Magnetic Resonance
OD_600_	Optical Density at 600 nm
PedA	Pediocin PA-1
SakP	Sakacin P
SPI	SUMO–Peptide–Intein
TFE	Trifluoroethanol
