# Supplementary material for: Solution Structure of the Broad-Spectrum Bacteriocin Garvicin Q
Source: Int J Mol Sci. 2025 Aug 14;26(16):7846. doi: 10.3390/ijms26167846 (PMC12386314; doi:10.3390/ijms26167846)
Supplement: Supplementary file 1 [file ijms-26-07846-s001.zip › ijms-3748769-supplementary.pdf]

Supplementary Information

"Solution Structure of the Broad-Spectrum Bacteriocin Garvicin Q."

Tyler Mallett, Tess Lamer, Tamara Aleksandrzak-Piekarczyk, Ryan T. Mckay, Karizza Catenza, Clarissa Sit, Jan K. Rainey, Kaitlyn M. Towle-Straub, John C. Vederas, and Marco J. van Belkum *

*Corresponding author: Department of Chemistry, University of Alberta, Edmonton, T6G 2G2, Canada. mvanbelkum@ualberta.ca

Table of Contents

1. **Supplementary Figures**……………………………………………………..……..S3-9

- His6-SUMO Isolation Procedure Diagram…………………………………….S3
- ^{15}N-HSQC for His$_6$SUMO…….……………………………………….……...S4
- CD Spectrum for LcnA………………………………………..……....…S5
- Ramachandran Plot GarQ…………………………………………..….S6
- Surface Hydrophobicity Plots ……………………………………………....S7
- Surface Electrostatic Plots……………………………………………..…S8
- Ramachandran Plot SUMO………………………………………..…..S9

2. **Supplementary Tables** ..……………………………………...…………..S10-13

- Amino Acid Sequences………………………………….……………….S10
- GarQ NMR Experimental Parameters……………………….……………..S11
- His$_6$-SUMO NMR Experimental Parameters……………….……………...S12
- His$_6$-SUMO Structural Statistics…………………………………………...S13

1. Supplementary Figures

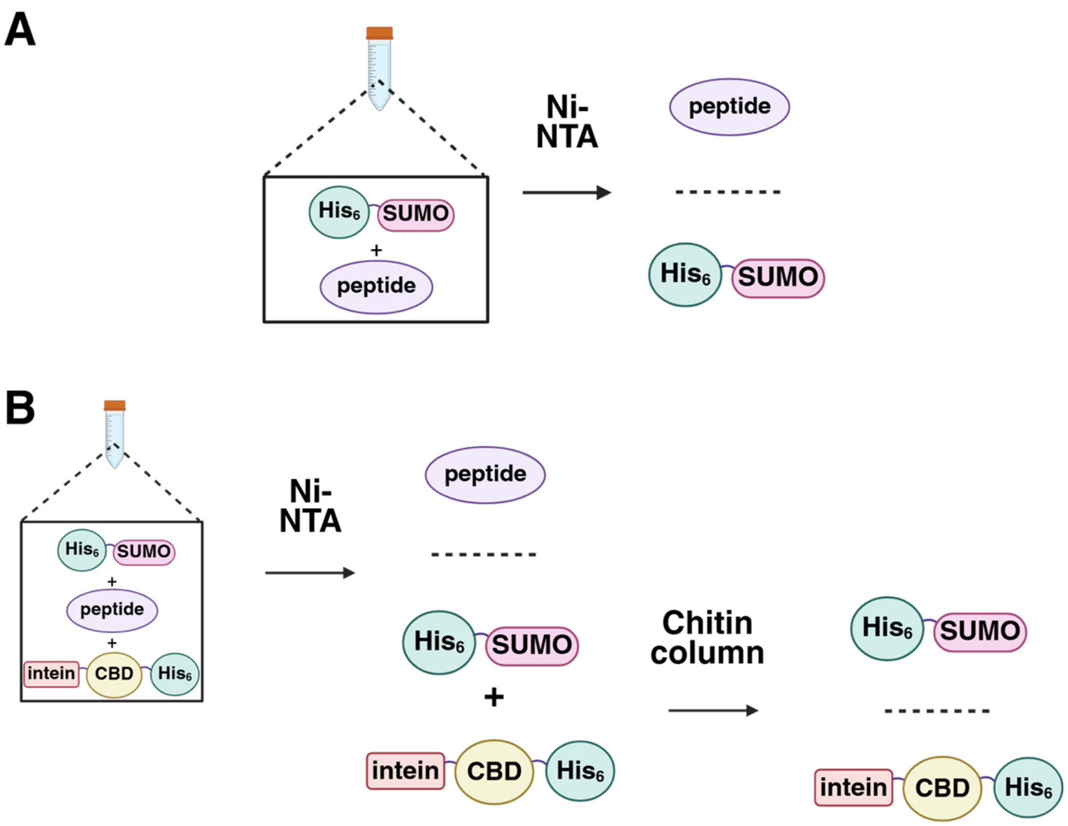

Figure S1: Cartoon representation of the His_6-SUMO isolation procedure. **(A)** is a representation of the post-affinity chromatography isolation of the desired peptide. **(B)** depicts the simple work flow to isolate His_6-SUMO from the intein-CBD-His_6 fusion protein.

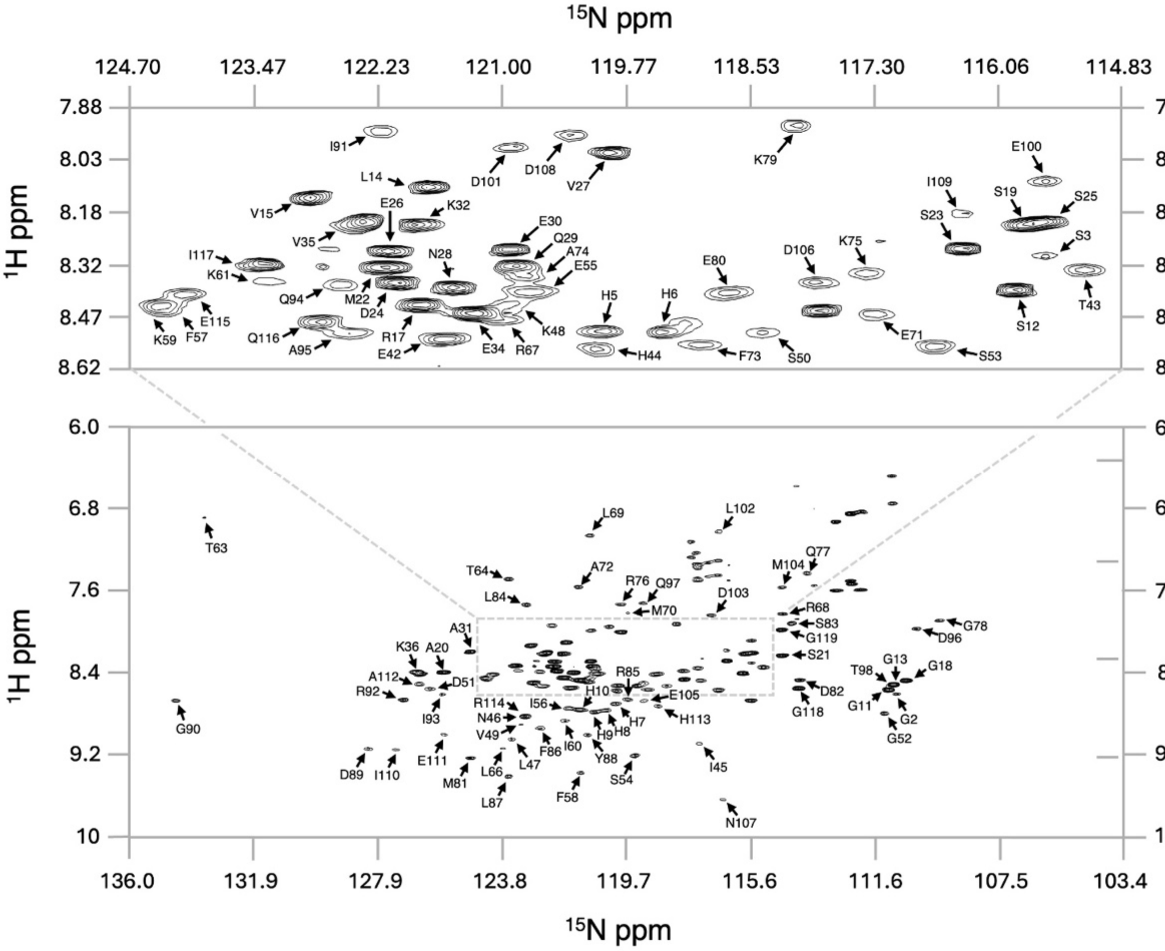

Figure S2: ^{15}N-HSQC spectrum of His$_6$-SUMO. Assigned full spectrum (bottom) and expansion of a central region (top). Assignment labels highlight each amino acid's amide NH chemical shift.

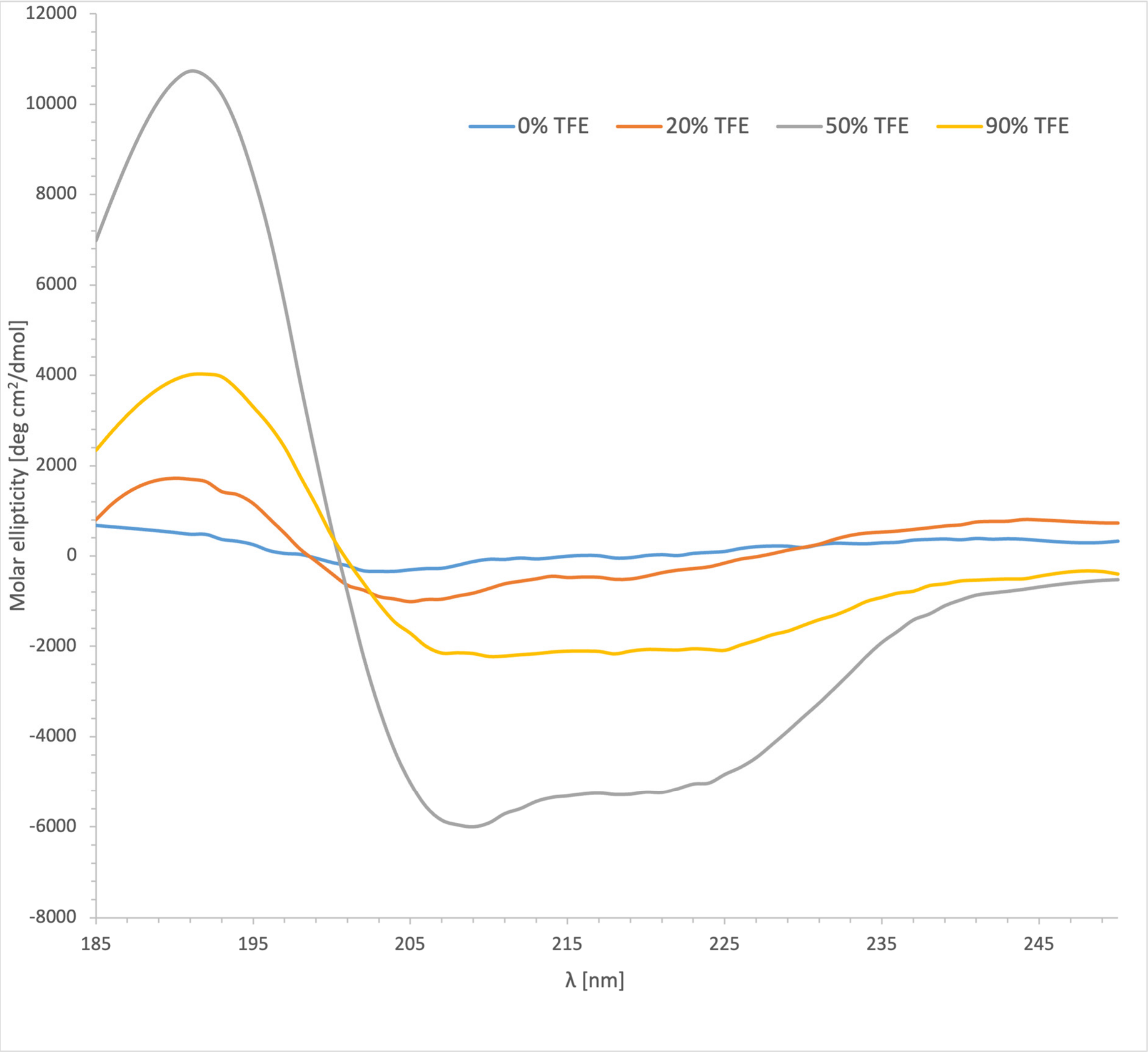

Figure S3: CD spectroscopic results of an unlabelled LcnA sample in differing TFE:H_2O solvent conditions. LcnA CD sample concentration = 0.5 mg/mL.

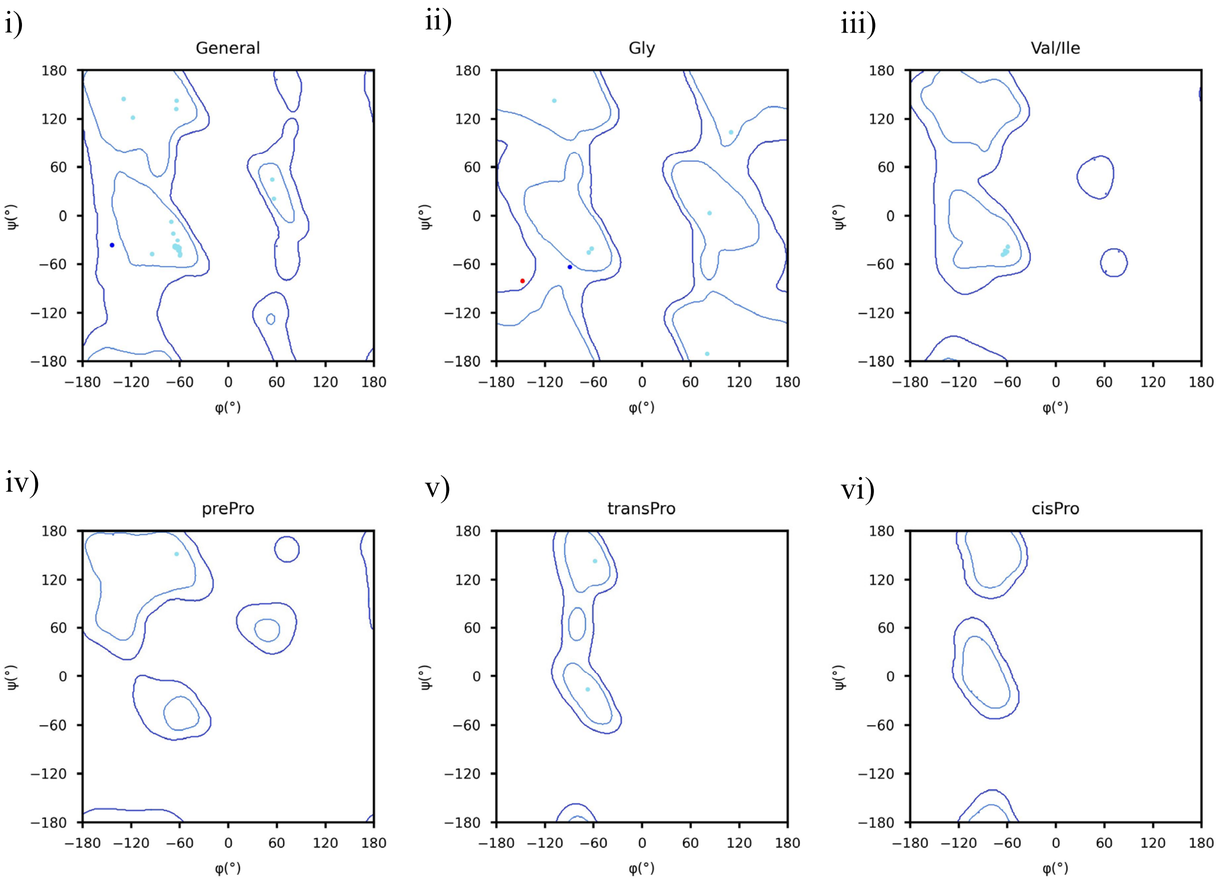

Figure S4: Ramachandran plots for the calculated GarQ NMR solution structure. Each peak represents an individual amino acid (Ψ, Φ) torsion angle. Cyan = favoured, royal blue = allowed, red = disallowed. **(i)** Ramachandran plot of all amino acids in the lowest energy structure. **(ii)** Glycine torsion angles only, disallowed data point = glycine 43. **(iii)** Valine and Isoleucine torsion angles only. **(iv)** Residues which precede a proline residue in the amino acid sequence. **(v)** Trans-proline torsion angles only. **(vi)** Cis-proline torsion angles only.

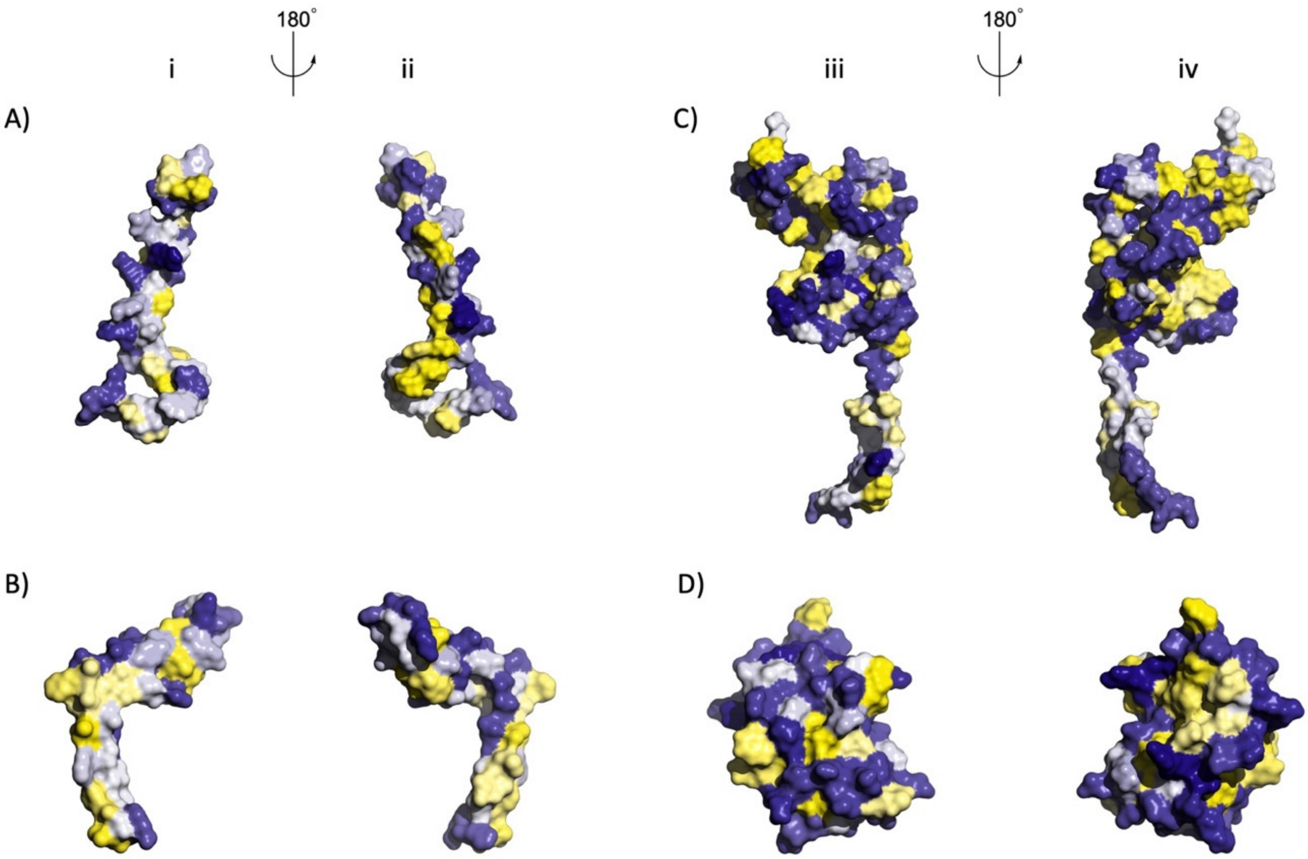

Figure S5: Surface hydrophobicity representation of the **(A)** GarQ NMR solution structure, **(B)** LcnA Cryo-EM structure, **(C)** His_6-SUMO lowest energy NMR solution structure, and **(D)** SUMO/Smt3 NMR solution structure. Hydrophobicity values were assigned based on the Kyte-Doolittle hydrophobicity scale, with yellow colouring representing hydrophobic regions.

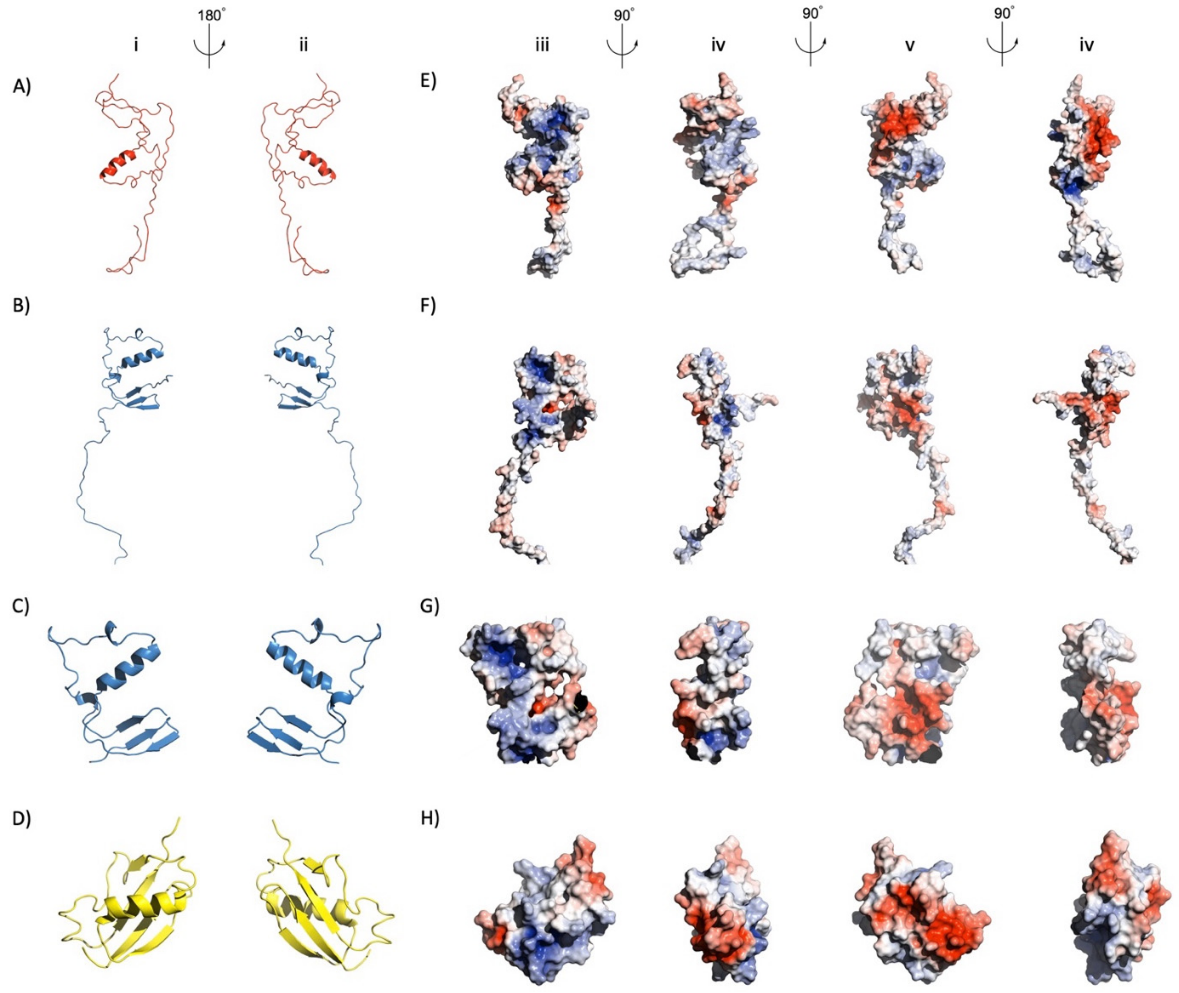

Figure S6: Structural model representations of **(A)** the lowest energy His$_6$-SUMO NMR solution structure, **(B)** His$_6$-SUMO low-energy structure 53, **(C)** His$_6$-SUMO low-energy structure 53 with N-terminal residues 1-41 missing, **(D)** Smt3/SUMO NMR solution structure with N-terminal residues 1-20 missing. In all cartoon images, the C-terminus is positioned at the top of the structure. Electrostatic surface representations of **(E)** the lowest energy His$_6$-SUMO NMR solution structure, **(F)** the His$_6$-SUMO low-energy structure 53, **(G)** His$_6$-SUMO low-energy structure 53 with N-terminal residues 1-41 missing, **(H)** Smt3/SUMO NMR solution structure with N-terminal residues 1-20 missing. Structures in columns **(i)** and **(iii)** share the same orientation, as do those in columns **(ii)** and **(v)**. Positively charged surface areas are shown in blue, negatively charged areas are shown in red, and neutral regions are shown in grey. Arrows and angle indicators show the axes and directions of rotation between views.

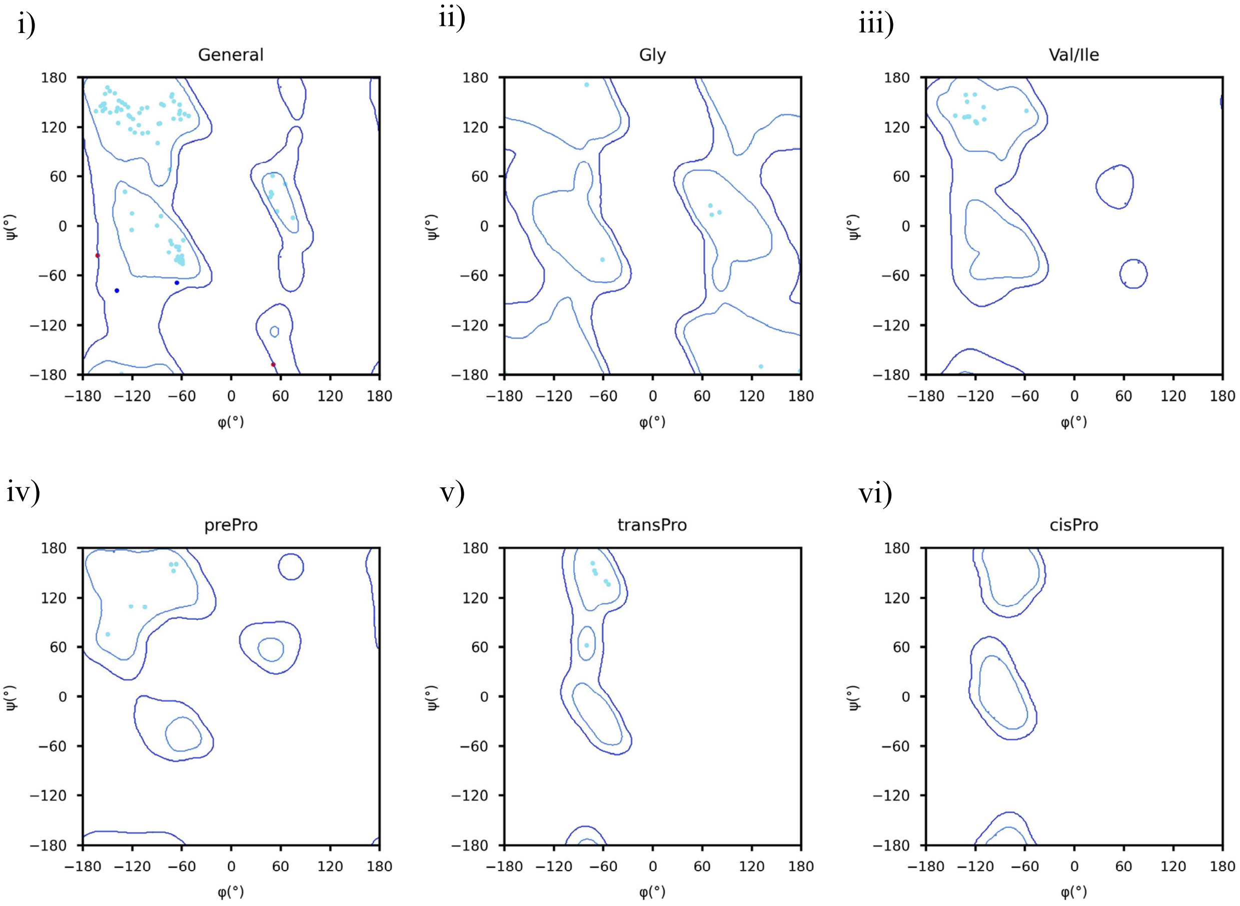

Figure S7: Ramachandran plots for the calculated His_6-SUMO NMR solution structure. Each peak represents an individual amino acid (Ψ, Φ) torsion angle. Cyan = favoured, royal blue = allowed, red = disallowed. **(i)** Ramachandran plot of all amino acids in the lowest energy structure. Disallowed residues are arginine 17 and lysine 62. **(ii)** Glycine torsion angles only. **(iii)** Valine and Isoleucine torsion angles only. **(iv)** Residues which precede a proline residue in the amino acid sequence. **(v)** Trans-proline torsion angles only. **(vi)** Cis-proline torsion angles only.

2. Supplementary Tables

Table S1: Amino acid sequences and monoisotopic masses for each peptide and protein used in this study.

Protein	Amino Acid Sequence	Predicted monoisotopic mass (^{13}C,^{15}N-enriched)	Observed monoisotopic mass (^{13}C,^{15}N-enriched)
GarQ	EYHLMNGANGYLTRVNGKYV YRVTKDPVSAVFGVISNGWGS AGAGFGPQH	5650.7 Da	5640.0 Da
His_6-SUMO	MGSSHHHHHH GSGLVPRGSA SMSDSEVNQEAKPEVKPEVKP ETHINLKVSDGSSEIFFKIKKTT PLRRLMEAFAKRQGKEMDSLR FLYDGIRIQADQTPEDLDMEDN DIIEAHREQIGG	14160.4 Da	14002.5 Da
		Predicted monoisotopic mass (^{13}C,^{15}N-natural abundance)	Observed monoisotopic mass (^{13}C,^{15}N-natural abundance)
LcnA	KLTFIQSTAAGDLYYNTNTHKY VYQQTQNAFGAAANTIVNGWM GGAAGGFGLHH	5778.4 Da	5784.5 Da

Table S2: Experimental parameters for each NMR experiment used to elucidate the solution structure for GarQ. [a] The nucleus acquired for each dimension (x for proton, y for carbon, z for nitrogen for the HNCACB experiment). [b] The number of transients squired for each FID. [c] The x,y,z pts is the number of points acquired in each dimention, sw is the sweep width for each respestive dimension where x is the directly detected dimension. [d] Mixing times are given in milliseconds.

experiment name	nuclei[a]	nt[b]	x-pts[c]	y-pts	z-pts	x-sw	y-sw	z-sw	mix[d]
^{15}N-HSQC	^{1}H,^{15}N	4	1786	64		10000	1800		
HNCACB	^{1}H,^{13}C,^{15}N	8	1786	64	32	10000	12319	1320	
^{15}N-HSQC-TOCSY	^{1}H,^{13}C,^{15}N	4	1786	128	32	10000	10000	1700	50
^{15}N-NOESY-HSQC	^{1}H,^{13}C,^{15}N	4	1786	128	32	10000	10000	1700	150
^{13}C-HcCH-TOCSY	^{1}H,^{13}C,^{15}N	8	1786	128	32	10000	10000	14080	11
^{13}C-NOESY-HSQC	^{1}H,^{13}C,^{15}N	16	1430	128	32	8390	7799	12070	120

Table S3: Experimental parameters for each NMR experiment used to elucidate the solution structure for His_6-SUMO. [a] The nucleus acquired for each dimension (x for proton, y for carbon, z for nitrogen). [b] The number of transients acquired for each FID. [c] The x,y,z pts is the number of points acquired in each dimension, sw is the sweep width for each respective dimension where x is the directly detected dimension. [d] Mixing times are given in milliseconds.

Experiment name	nuclei [a]	nt [b]	x-pts [c]	y-pts	z-pts	x-sw	y-sw	z-sw	Mix [d]
^{15}N-HSQC	$^{1}H,^{15}N$	4	1430	64		8390	2000		
HNCA	$^{1}H,^{13}C,^{15}N$	8	1430	96	32	8390	4526	2000	
HN(CO)CA	$^{1}H,^{13}C,^{15}N$	16	1430	128	32	8390	4526	2000	
CBCA(CO)NNH	$^{1}H,^{13}C,^{15}N$	16	1704	64	32	10000	12070	2188	
HNHA	$^{1}H,^{15}N$	24	1430	64	32	8390	4000	1800	
HNCACB	$^{1}H,^{13}C,^{15}N$	24	1430	64	32	8390	10561	1824	
HNCO	$^{1}H,^{13}C,^{15}N$	8	1430	64	32	8390	3018	2000	
HN(CA)CO	$^{1}H,^{13}C,^{15}N$	48	1430	32	32	8390	3018	1800	
^{15}N-HSQC-TOCSY	$^{1}H,^{13}C,^{15}N$	16	1430	96	32	8390	6700	2000	50
^{15}N-NOESY-HSQC	$^{1}H,^{13}C,^{15}N$	16	1430	96	32	8390	7800	1800	120
^{13}C-HcCH-TOCSY	$^{1}H,^{13}C,^{15}N$	4	2048	40	60	12820	16077	6000	11
^{13}C-NOESY-HSQC	$^{1}H,^{13}C,^{15}N$	8	2048	40	100	12820	8652	9597	120

Table S4. Structural Statistics for His_6-SUMO. Data with an * is provided by Xplor-NIH calculation statistics. Unmarked data is provided by the wwPDB Structure Validation Service.

No. of constraints	
Total No. of NOE constraints	266
Short-range	138
Medium-range ($\mathbf{1 < \lvert i - j \rvert < 5}$)	36
Long-range ($\mathbf{\lvert i - j \rvert > 5}$)	25
Total No. of dihedral angle constraints	142
ϕ	58
ψ	58
χ	26
Constraint Violations	
Distance constraints	3
Dihedral-angle constraints	0
RMSD of the calculated protein structure	
*Whole structure	0.25 ± 0.07
A:65-A:92 (28) (Å)	1.96
Ramachandran statistics (%)	
Favoured regions	112
Allowed regions	3
Disallowed regions	2
Assignment statistics	
No. of assigned peaks	1159
No. of unassigned peaks	449
Assignment completeness	72%
